# Illusory shrinkage of objects under backward masking

**DOI:** 10.1177/20416695241304655

**Published:** 2024-12-05

**Authors:** Sae Kaneko, Stuart Anstis, Patrick Cavanagh

**Affiliations:** Department of Psychology, 12810 Hokkaido University, Sapporo, Japan; Department of Psychology, University of California San Diego, La Jolla, CA, USA; Department of Psychology, Glendon College, North York, ON, Canada; CVR, 197332 York University, North York, ON, Canada; Psychological and Brain Sciences, Dartmouth College, Hanover, NH, USA

**Keywords:** spatial vision, masking, size perception

## Abstract

Backward masking is a powerful phenomenon that can reduce, often to zero, the visibility of targets. Here, we show that when the masking is less than completely effective so that the target remains visible, the masking has other effects, specifically reducing the perceived size of the target.

The perceived size of an object does not depend only upon the amount of retinal area that it covers; for example, an object's apparent size can be drastically changed by its perceived distance. But here we refer to much earlier visual processes, related to masking, that also affect perceived size. Movie 1 presents two identical red squares, each inside an identical white box. The upper and lower red squares are presented for the same duration of 50 ms and the only difference between them is in the duration of the surrounding box. The upper square and its box both turn off after 50 ms, whereas the lower box remains on for an additional 200 ms after the lower red square is extinguished (the lower box is on for a total of 250 ms). Surprisingly, the lower red square looks noticeably smaller than the upper one.

**Movie 1.** While looking anywhere but directly at the flashing red squares, for example, at the black dot on the right, make a judgment of the relative sizes of the top and bottom squares. The bottom square may look smaller. Both red squares are presented for the same 50 ms but the top, surrounding white box is present only for 50 ms but the bottom one remains on for another 200 ms (250 ms total). The lingering white box on the bottom produces partial backward masking or object substitution masking of the lower red square that affects its apparent size without yet rendering it invisible.

This effect of the lingering surround is quite likely related to masking, specifically, object substitution masking (Enns, 2004; Enns & Di Lollo, 1997, 2000). These authors found that four dots arranged in a notional square could strongly mask an object presented in peripheral vision at an unattended location. Moreover, when dots appeared beside a briefly presented target object, and persisted in view longer than the target, the dots could alter the flanked object's shape by deleting parts of it (Kahan & Enns, 2010). In contrast to these earlier effects of object substitution masking, our stimuli could be viewed in central vision and did not have to be unattended. The effect in Movie 1 was not to reduce the visibility or contrast of the target nor change its shape by deleting parts of it. Instead, the backward masking of the red square simply decreased its apparent size.

Next, we asked if there was a critical timing for the appearance of the target within the border. Movie 2 shows that the perceived size of the briefly presented red squares on the bottom row is affected most if it appears early in the interval when the surrounding box is present. All four white boxes are on for 550 ms. The top red square remains on for the full 550 ms along with its box to serve as a control showing the veridical size of all the red squares. In the bottom row, all three red squares appear for only 50 ms, but the leftmost one appears at the same time as the surrounding box, the middle one appears in the middle of the 550 ms, and the right hand one at the end of the 550 ms. The apparent size of the squares, reading from left to right, may look small, medium, and large when fixating on the black dot on the right. So, for the maximum amount of size compression the test red square should come on simultaneously with the frame, not later, consistent with maximum backward masking.

**Movie 2.** Here the top red square is presented for 550 ms along with its surrounding box. It serves as the control to show the actual size of all the red squares. On the bottom, the boxes are also presented for 550 ms while the three red squares are presented briefly (50 ms) either at the beginning (left), in the middle (middle) or at the end (right) of the presentation of the surrounding box. The size of the red squares on the bottom, although all identical, may appear small, medium, and large from left to right, respectively, while fixating the black dot on the left. The leftmost version is most consistent with backward masking and produces the largest effect.

In sum, backward masking can suppress the visibility of targets (Breitmeyer, 1984). However, here, we show that when the masking is less than completely effective, the target may remain visible but be reduced in size.
